# Supplementary material for: The causal relationship between the human gut microbiota and pyogenic arthritis: a Mendelian randomization study
Source: Front Cell Infect Microbiol. 2024 Nov 26;14:1452480. doi: 10.3389/fcimb.2024.1452480 (PMC11629706; doi:10.3389/fcimb.2024.1452480)
Supplement: Supplementary file 1 [file DataSheet1.zip › mendelian test/ebi-a-GCST90027547.csv_leave_one_out.pdf]

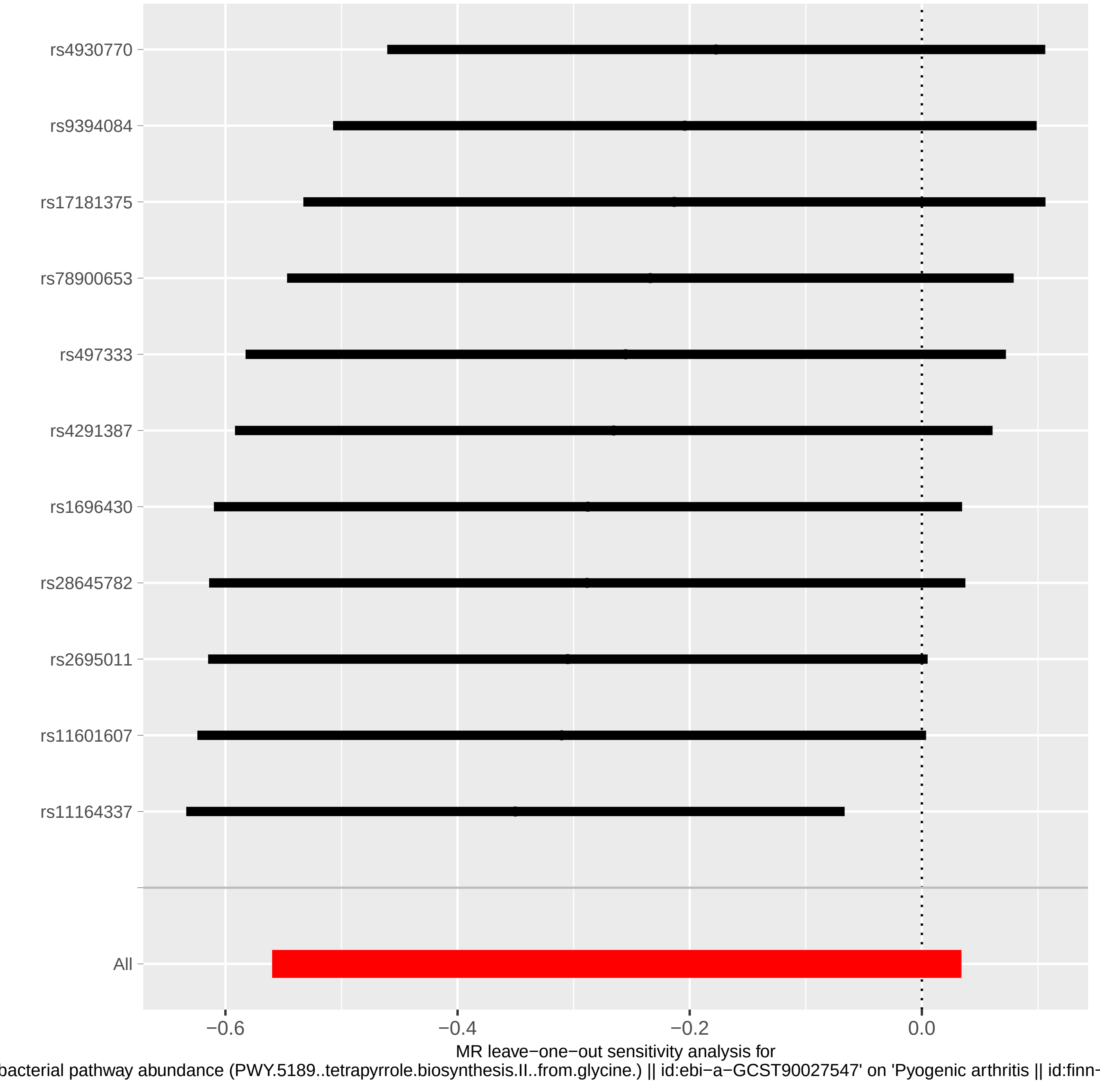

rs4930770
rs9394084
rs17181375
rs78900653
rs497333
rs4291387
rs1696430
rs28645782
rs2695011
rs11601607
rs11164337
All
−0.6
−0.4
−0.2
0.0
MR leave−one−out sensitivity analysis for
bacterial pathway abundance (PWY.5189..tetrapyrrole.biosynthesis.II..from.glycine.) || id:ebi−a−GCST90027547' on 'Pyogenic arthritis || id:finn−
